# Supplementary material for: Single V2 defect in 4H silicon carbide Schottky diode at low temperature
Source: Nat Commun. 2025 May 20;16:4669. doi: 10.1038/s41467-025-59647-9 (PMC12092576; doi:10.1038/s41467-025-59647-9)
Supplement: Supplementary file 1 — Supplementary Information [file 41467_2025_59647_MOESM1_ESM.pdf]

Supplementary Information: Single V2 defect in 4H Silicon Carbide Schottky diode at low temperature

Timo Steidl,[1,*] Pierre Kuna,[1,*] Erik Hesselmeier-Hüttmann,[1] Di Liu,[1,2] Rainer Stöhr,[1] Wolfgang Knolle,[3] Misagh Ghezellou,[4] Jawad Ul-Hassan,[4] Maximilian Schober,[5] Michel Bockstedte,[5] Guodong Bian,[6,7,8] Adam Gali,[6,7,8] Vadim Vorobyov,[1,†] and Jörg Wrachtrup[1,9]

[1] *3rd Institute of Physics, IQST, and Research Center SCoPE, University of Stuttgart, Stuttgart, Germany*
[2] *John A. Paulson School of Engineering and Applied Sciences, Harvard University, Cambridge, MA 02138, USA*
[3] *Department of Sensoric Surfaces and Functional Interfaces, Leibniz-Institute of Surface Engineering (IOM), Leipzig, Germany*
[4] *Department of Physics, Chemistry and Biology, Linköping University, Linköping, Sweden*
[5] *Institute for Theoretical Physics, Johannes Kepler University Linz, Linz, Austria*
[6] *HUN-REN Wigner Research Centre, Budapest, Hungary*
[7] *Institute of Physics, Department of Atomic Physics, Budapest University of Technology and Economics, Budapest, Hungary*
[8] *MTA-WFK Lendület "Momentum" Semiconductor Nanostructres Research Group*
[9] *Max Planck Institute of Solid State Research, Stuttgart, Germany*

I. SUPPLEMENTARY NOTE 1: MEASUREMENTS OF PLE LINEWIDTH

The voltage we can apply for Stark tuning or linewidth narrowing is limited by the I-V-characteristics (Supplementary Fig. 1a & Fig. 1d in the main text) of the diode. In reversed bias we have nearly no limit as no or just very little reversed current is created. For positive voltages, the threshold voltage luckily rises for lower temperature, that gives a larger voltage range for forward voltage bias at cryogenic temperatures to operate without sample heating and increasing the linewidths caused by current flow. Too large current will also cause electroluminescence (see Supplementary Fig. 5) or start degradation of the contacts.

As we are using the two-laser PLE scheme for all measurements, we can not extract both single linewidths for A_1 and A_2, instead we see a superposition of both linewidths. Both processes sum up and as the A_2 transition is typically more dominant, we would expect a PLE linewidth in the same range if we approach the Fourier limit. For the calculations of the lifetime limits used in the main figure Fig. 2b, where are using the equation $\Delta\nu = 1/(2\pi\tau_r)$. For the excited state lifetimes of $A_{1,2}$ we get $\tau_{A_{1,2}} = 1/(\gamma_r + \gamma_{1,2} + \gamma'_{1,2}) = 6.09|11.35\,\text{ns}$ using the values from Ref. [1]. From that we derive $\Delta\nu_{A_{1,2}} = 26.1|14.0\,\text{MHz}$ as Fourier limits. To investigate further the natural linewidth of the defect in the depleted area we measure ple linewidth power dependence. We measured the PLE linewidth versus resonant laser power for two voltages. For the depleted case we typically get an extrapolated linewidth of 40 MHz. This is already quite close and might be related to some remaining charges as we also found a defect approaching the lifetime limit (main text) If we apply positive voltage we get additional broadening from the inserted charges, which offsets the curve in Supplementary Fig. 2b. An additional step-like behaviour is visible, which might indicate that even a small amount of resonant light leads to increased charge mobility and subsequent broadening of the PLE lines around the defect.

Not only bulk defects has been studied this work, also defects integrated into 1 µm wide nano beams. Supplementary Fig. 1b show the full confocal scan of Fig. 1e in the main text, here, some of the one-sided tapered waveguides can be seen. So, we show PLE measured in a waveguide and observed line shifts from the Stark effect as well. Each section in Supplementary Fig. 1c, between the grey dashed lines, are subsequent PLE scans having the same bias voltage applied, which is labeled on the left. Smooth voltage scans like in the main text could not be done as the defect would drift faster than the Stark shift would do. Using such big voltage steps make the Stark effect quite visible but also drifting and jumping of the PLE lines is still quite recognisable. The absolute lineshift is also larger than for bulk defects with similar or even shorter distance to the stripline. This can be explained as the electric field is screened less, as it is propagating through fewer charge containing material. As the Schottky contact is to far ($> 30\,\mu\text{m}$) from the waveguides we did not observe any linewidth narrowing or stabilisation. Apparently, we might no be able so see narrowing in nano beams at all as color centers appear already less broad with linewidths of $<$30 MHz approaching the Fourier limit ($\sim$ 20 MHz) (compare Supplementary Fig. 1d). We attribute this to the lack of noticeable volume charge which can lead to broadening. On the other hand the defect is closer to the surface of the beam, thus making it more sensitive to surface charges despite the fact that it is still more stable than e.g. NV centers close to the surface. This could be an indicator for that SiC is a more comfortable host material as it might has less

* These two authors contributed equally
† v.vorobyov@pi3.uni-stuttgart.de

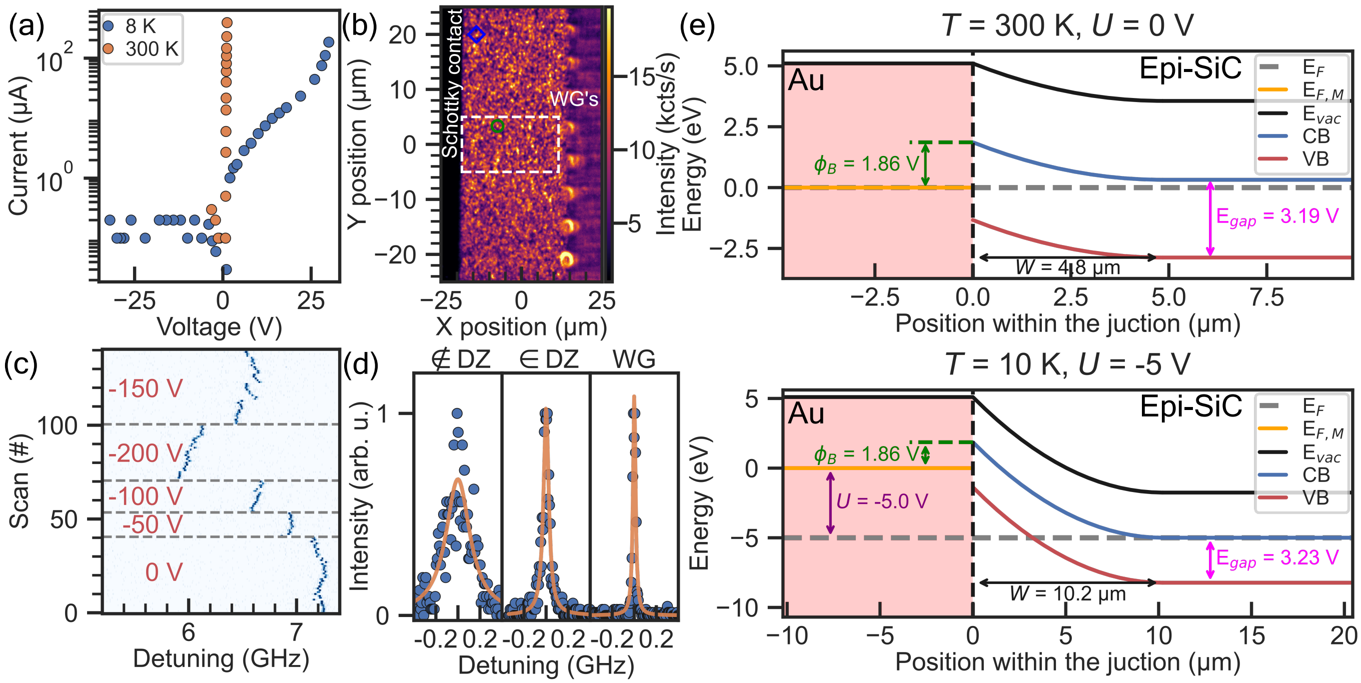

SUPPLEMENTARY FIGURE 1. **(a)** Diode characteristics for two different temperatures between room and cryogenic temperatures, also showing the small reverse saturation current. **(b)** Full confocal map showing the position of the defects from the statistics in Fig 2 in the main text (dashed white rectangular) and location of the waveguides. The defect-1 (green circle) and defect-2 (blue diamond) were used for the repetitive PLE measurement in Fig 2a. **(c)** Subsequent PLE measurements of the V2 centre in a waveguide. Each block has fixed applied bias voltage and the jumps at the boundaries are caused by Stark shift as we change the voltage and thus also the electric field. Within each block the voltage is kept constant, so every jump or drift is related to surface charge fluctuations. Higher negative voltage seems not to improve defect stability. **(d)** Comparsion of PLE measurement of defects within ($\Delta\nu_{\in} = 56.7\pm1.3\,\mathrm{MHz}$) and without ($\Delta\nu_{\notin} = 267.4\pm14.3\,\mathrm{MHz}$) the depletion zone as well as integrated into a nanophotonic beam. PLE narrowing by depletion is clearly shown. Suprisingly, defects in waveguides appear even more narrow ($\Delta\nu_{\mathrm{WG}} = 27.2\pm0.5\,\mathrm{MHz}$). **(e)** Simplified energy band diagram of the Schottky interface for abient conditions (upper panel) and at cryogenic temperatures with additional negative bias voltage (lower panel).

surface charges in total.

In Supplementary Fig. 2a one can see the alternating PLE position and switching to broad linewidth for near zero voltages. In total we swept the voltage 3 times each back and forth between +20 V and -150 Volts. Also the repeatability with almost no hysteresis is remarkable but not given for every defect or high repump laser power. The data presented in the Fig 2b of main text is extracted from that entiry PLE scan. The values from single line fits were averaged over multiple voltage sweeps. One might notice some missing data points. In these lines the defect was ionised and not successfully repumped. It is also notable to say, that the PLE position seems to be tuned in the opposite direction as soon as it is outside of the depletion zone. At the edge of the depletion zone some of the charges outside want to go inside again to form equilibrium state and this will cause a build-in field counteracting the one from the contact. The defect will then probably be located in between and react according to the sign switching of the electric field.

II. SUPPLEMENTARY NOTE 2: NUMERICAL SIMULATION OF THE DEPLETION ZONE

As in the main text mentioned the simulation of the edge of the depletion zone has been optimised to match with the data of the PLE statistics. In the COMSOL simulation we swept the initial intrinsic layer doping concentration as well as the studied defect location depths. In Supplementary Fig. 2c & d it is shown how the depletion zone would occur using the other parameters. Partly the difference is not that significant but some values can be excluded as it would cause a different shape. It is worth to notice again that for each line of a certain distance multiple defect have been used and averaged, depending on the amount of emitters inside a confocal spot and PLE scan range. The bars of each line have been expanded or narrowed upon the next laying line to close potentially gaps in the data set. Supplementary Fig. 2e nicely shows the evolution of the depletion zone for different temperatures and voltages. Even at room temperature a large area inside the epi-layer is depleted under the metal contact. By going down to cryogenic temperatures this area grows and get well defined edges. The lower the temper-

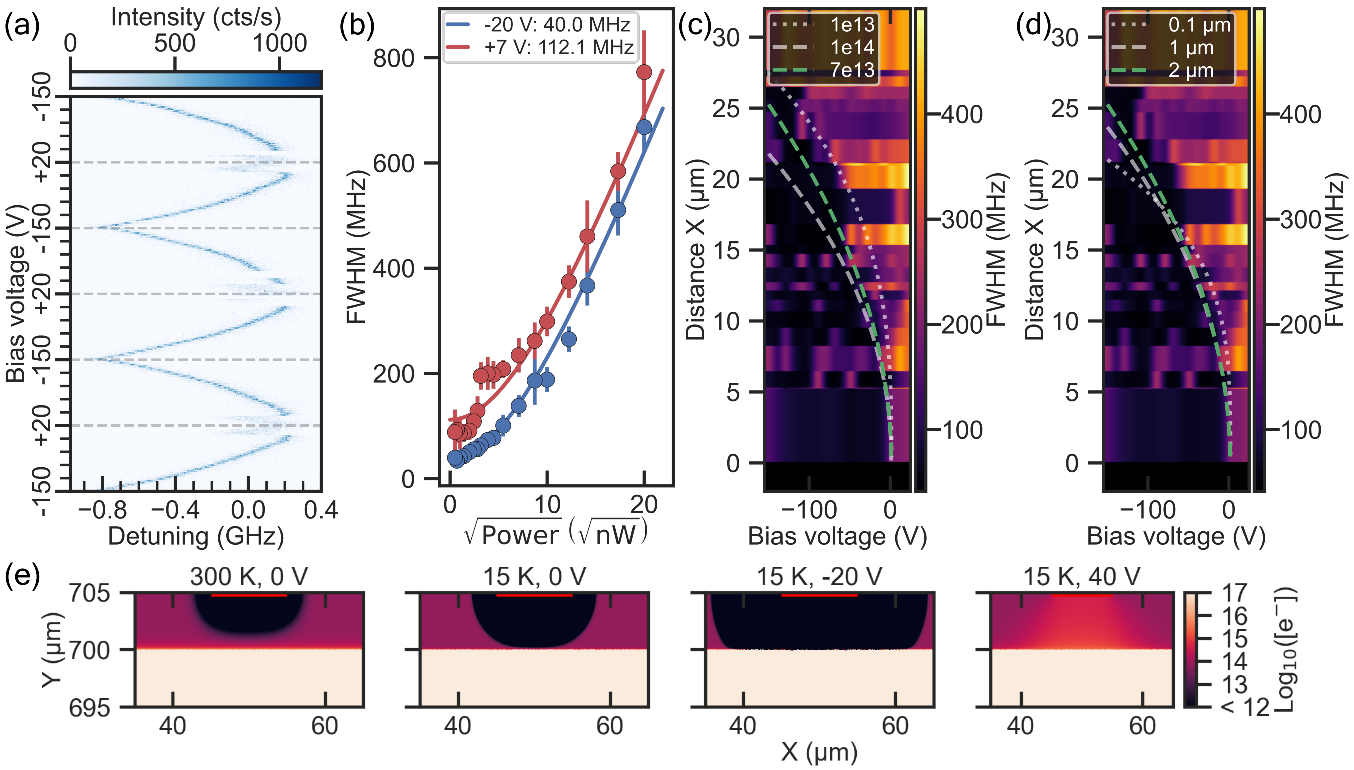

SUPPLEMENTARY FIGURE 2. **(a)** Repeated voltage sweeps during PLE (defect-1) used for Fig 2a in the main text and show reprocudability. The detuning of all PLE's in sample A are relative to a resonant laser frequency of 327.123 THz, 327.116 THz for sample B, respectively. **(b)** PLE linewidth broadening for two bias voltages (error bars $\widehat{=} \pm 1\sigma$). **(c)** Depletion zone mapping for different initial epi dopings, $[N]$ = 1e13, 7e13 & 1e14 cm^{-1}. **(d)** Depletion zone mapping for different defect depths: 100 nm, 1 μm & 2 μm **(e)** COMSOL [2] simulation of the carrier density for different temperatures and voltages. Red line shows contact boundary to the electrode.

ature the smaller are the fluctuations, so the simulation done for 15 K also holds for the 8 K, the temperature which has been used for most of our measurements. Increasing the negative bias shows the same effect. Even if it is not visible in this plot, there are also some charges removed from the wafer side, if the temperature is low or the reversed voltage high enough. But this effect is so small that it does not make an interesting change, the counteracting electric field is also higher there, stopping electrons further to be removed. It is also shown, that for positive voltages this depletion zone does not only vanish, we also insert additional charges into the epi-layer, which enables current flow and thus could lead to heating and spectral broadening of the optical lines. Some details of the setup for the COMSOL simulations are shown in Supplementary Tab. I.

SUPPLEMENTARY TABLE I. Used paramter values of the simulation of depletion zone and electric field:

Parameter (@15 K)	Value	Unit
Workfunction (Au)	5.1	eV
Workfunction (Ti)	4.5	eV
Band gap (4H-SiC)	3.23	eV
Refractive index	2.588	1
Electron affinity	3.24	eV
Doping concentration (epi)	7e13	cm^{-3}
Doping concentration (wafer)	1e17	cm^{-3}
Eff. density of states, VB	2.7885e23	m^{-3}
Eff. density of states, CB	1.8881e23	m^{-3}
Electron mobility (epi)	237.02	$m^2V^{-1}s^{-1}$
Electron mobility (wafer)	29.556	$m^2V^{-1}s^{-1}$
Effective mass (e)	3.6578e-31	kg
Effective mass (h)	2.4083e-30	kg

III. SUPPLEMENTARY NOTE 3: CHARGE STABILITY AND ELECTROLUMINESCENCE

Large off-resonantly excited confocal scans have been taken at different bias voltages to show there is no difference in respect to each other, also by subtracting them no clear difference can be observed (Supplementary Fig. 3). It seems the defect are enterly charge stable as no V2 appears or disappears. This is a contrary result to what has been observed previously in [3] at room temperature in a PIN diode.

Supplementary Fig. 4 shows additional information for sample 2. There, confocal maps of the electrode geom-

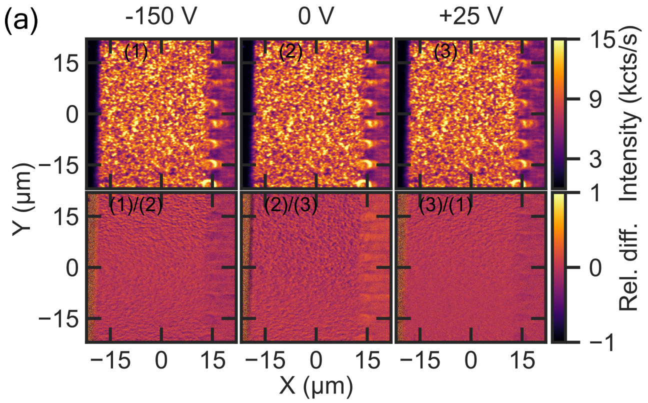

SUPPLEMENTARY FIGURE 3. **(a)** Comparsion of confocal scans at three different bias voltages ($-150\,\mathrm{V}/0\,\mathrm{V}/25\,\mathrm{V}$). Lower row show subtracted measurements. No significant change can be identified, so so defects appear or disappear by changing the voltage, leading to the assumption, that the defects are charge stable within the voltage range and for off-resonant excitation.

etry as well as of each SIL for every defect used in this paper. The voltage is usually applied between one of the top gold contacts (1-3) and the bottom of the sample connected to GND. A typical diode characteristics of contact 2 is shown in Supplementary Fig. 4e for three temperatures. For negative bias PLE Stark shift and linewidth narrowing down to 40 MHz can be observed (see Supplementary Fig. 4f & g), showing similar behaviour to previous measurements. From the main text, the spin coherences at -30 V for different DD sequences are normalised to their number of refocussing pulses in Supplementary Fig. 4f. Here, small deviations in from theoretical $\propto N^{2/3}$ dependency and fluctuations of the strechting exponent causing the non-identical lines. For the sake of completeness, we also measured the spin coherence of one nuclear spin. For the 5 MHz coupled ^{13}C nuclear spin we observe a T_2 time of 63±4 ms.

As we can send current through a semiconductor host material if we pass the threshold voltage, we expect electro luminescence response of the material. And indeed for forward voltages higher than 19 V we measure a diode current of several tens of milliampere. Besides of the rising base temperature of the cryo (increase by a few Kelvin), we observe a rising detected photon count rate on the photodetectors (SNSPD) with all laser sources being switched off. The electroluminescence is strong enough that it is visible by eye as a small white light source inside of the cryotation (observable through a side window, see picture in Supplementary Fig. 5a). It seems that the light is almost emitting purely lateral, as it can be observed from the window but not from the objective on top. Only on the curved surface of the SILs some light can be detected (compare Supplementary Fig. 5b upper and lower part), as there will be an appropriate angle to reflect the light upwards. The electroluminescence intensity seem to grow linearly with the current (or exponentially with the voltage, Supplementary Fig. 5d). Interestingly, we also observed hysteresis on a rather large voltage range. In contrast to the sharp rise of the current after passing the threshold, the breakthrough can be maintained almost to half the breakthrough voltage on the way back to 0 V (dark blue line in Supplementary Fig. 5c), presumably due to increased local temperature. With two Ocean Optics spectrometers for different wavelength ranges (as the emitted light unavoidable splits at a 800 LP filter) we observed the full spectra of the electroluminescence, shown in Supplementary Fig. 5e. It shows a very broad spectrum around 700 nm with a long tail into the near-infrared.

IV. SUPPLEMENTARY NOTE 4: THEORETICAL APPROACH OF PHOTO-IONISATION RATE

Here we outline the theoretical approach for the calculation of the photo-ionisation rate of the negatively charged cubic silicon vacancy $\mathrm{V}_{\mathrm{Si}}^-$ shown in Fig. 3d of the main text. In our experiments the defect is photo-ionised by a two photon process. The first photon resonantly excites the ground state to the first excited quartet state and subsequent photon with energy $h\nu$ from the laser with variable wavelength ionises the vacancy. In Supplementary Fig. 6a we depict the process in which a doubly negative vacancy and a hole are produced, $\mathrm{V}_{\mathrm{Si}}^- \to \mathrm{V}_{\mathrm{Si}}^{2-} + h^+$. We calculate the ionisation cross section from the resonantly excited state following [5]. While the initial state $|\Psi_{\mathrm{ex}}\rangle$ is resonantly excited from ground state and therefore does not contain any vibrational quanta, the final states $|\psi_{f,vib}\rangle$ describe the vibrationally excited ionised vacancy $\mathrm{V}_{\mathrm{Si}}^{2-}$ and a hole in the valence band. Within the Huang-Rhys theory, the cross section for the purely electronic transition between $|\Psi_{ex}\rangle$ and electronic part of the ionised states $|\Psi_f\rangle$ is given by

$$\sigma_{\mathrm{PI}}^{\mathrm{el}}(\varepsilon) = \frac{4\pi^2\alpha}{3\,n_D}\varepsilon \sum_{ex,f} |\langle\Psi_{ex}|\hat{\vec{r}}|\Psi_f\rangle|^2 \delta(\varepsilon - \Delta E) \quad , \qquad (1)$$

where $\Delta E = E_f - E_{ex}$, α is the fine-structure constant, and n_{D} is the refractive index of 4H-SiC. The sum includes all spin substates of the first excited states and the final states with holes in the valence band that are compatible with the photon energy $\varepsilon = h\nu$. Vibrational effects are incorporated into the full cross section $\sigma_{\mathrm{PI}}(\varepsilon)$ by convolution of $\sigma_{\mathrm{PI}}^{\mathrm{el}}(\varepsilon)$ with the vibrational spectral function $A(\varepsilon)$ in the following way (cf. e.g. Ref. [5])

$$\sigma_{\mathrm{PI}}(\varepsilon) = \varepsilon \int_{\infty}^{\infty} \frac{1}{\varepsilon'}\, \sigma_{\mathrm{PI}}^{\mathrm{el}}(\varepsilon')\, A(\varepsilon - \varepsilon')\, d\varepsilon' \quad . \qquad (2)$$

The first step is the calculation of $\sigma_{\mathrm{PI}}^{\mathrm{el}}$. The states $|\Psi_{ex}\rangle$ and $|\Psi_f\rangle$ are correlated multiplet states and can be obtained by correlated first principles approaches such as our CI-CRPA method [3, 6]. Only in special cases, these

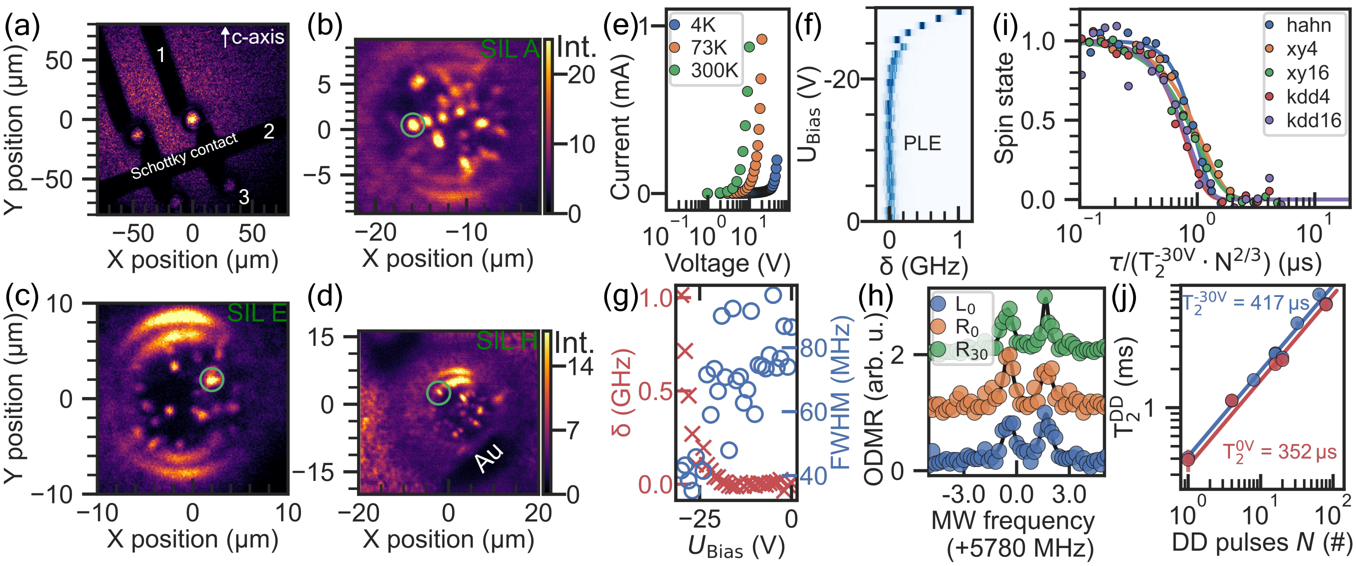

SUPPLEMENTARY FIGURE 4. More detailed description of sample B: **(a)** Multiple SILs and the surrounding electrical contact. **(b)** SIL A, showing the V2 center which is coupled to the 5 MHz nuclear spin. Used for the DD sequence measurements. **(c)** SIL E, showing the defect with three charge states. **(d)** SIL H, showing the defect with 2 charge states used for time transient plot. **(e)** I-V characteristics of contact 2 (in between the SILs) **(f)** Typical PLE scans under bias voltage for defect marked in in SIL A (b). **(g)** Fitted linewidth and PLE position from (f). **(h)** ODMR measured at both transitions (L: left, R: right) from defect in (d) (Figure 2e in the main text), showing that the PLE lines belong to the same defect. ODMR measured also at -30 V bias, showing that there is no voltage dependency. **(i)** To $\tau/\mathrm{T}_2(N)$ rescaled DD sequences, using the extracted values in (j) for -30 V. **(j)** Comparsion of coherence times of the DD sequences for 0 V and -30 V. Lines are fitted with $T_2(N){=}\mathrm{T}_2^i \cdot N^{2/3}$ characteristics [4].

states reduce to single slater determinants and are directly accessible in (constrained) density functional theory [6]. As the summation over the final states samples the k-space regarding the hole states and the convergence of this summation is demanding, the direct approach is computationally not feasible at present. Therefore we adopt a different approach and construct the initial and final multiplet states from ground state Kohn-Sham orbitals of $\mathrm{V}_{\mathrm{Si}}^{-}$ based on our CI-CRPA results for the cubic $\mathrm{V}_{\mathrm{Si}}^{-}$ and $\mathrm{V}_{\mathrm{Si}}^{2-}$. For instance, in the case of $M = \pm 3/2$ spin component of the initial first excited quartet (V2-line), the leading components ($\sim$94 %) are given by $|ue_xe_y\rangle$ and $|\bar{u}\bar{e}_x\bar{e}_y\rangle$ using the hole notation and the overbar indicating negative spin. The second photon excites a hole h ($\bar{h}$) into the empty u ($\bar{u}$) yielding $|he_xe_y\rangle$ ($|\bar{h}\bar{e}_x\bar{e}_y\rangle$), i.e. the triplet ground state of $\mathrm{V}_{\mathrm{Si}}^{2-}$ with a free hole $|^3A_2, M = -1\rangle \otimes |h\rangle = |e_xe_y\rangle \otimes |h\rangle$ ($|^3A_2, M = 1\rangle \otimes |\bar{h}\rangle = |\bar{e}_x\bar{e}_y\rangle \otimes |\bar{h}\rangle$). The transition matrix element in Eq. (1) becomes the matrix element between the Kohn-Sham states $|\langle h|\vec{r}|v\rangle|^2$, where we use the spin-orbitals from our spin-polarised calculation that are actually involved in theexcitation.

A similar analysis for the $M = \pm 1/2$ components of the first excited quartet, yields the same contributions to the cross section. The excited hole quartet state decomposes e.g. for $M = 1/2$ into $\sqrt{\frac{2}{3}}\,|^3A_2, 0\rangle \otimes |h\rangle$ and $\frac{1}{\sqrt{3}}\,|^3A_2, 1\rangle \otimes |\bar{h}\rangle$, i.e. components of the ground state triplet of $\mathrm{V}_{\mathrm{Si}}^{2-}$ with a hole in the valence band. The excitation energy $\Delta E = E_f - E_{\mathrm{ex}}$ is approximated by $\varepsilon(-|2-) - E_{\mathrm{V2}} - \Delta E_h$, where $\varepsilon(-|2-)$, E_{V2} and ΔE_h are the threshold for ionisation between the ground states of the two charge states with respect to the valence band edges, the transition energy of the $V2$ line, and the energy separation of the hole state from the valence band edge. For $\varepsilon(-|2-)$ we use the value from [3] obtained by hybrid DFT (see Fig. 3c). E_{V2} is given by the experimental value which is well approximated by constrained DFT, and ΔE_h is taken from the DFT-PBE calculations (see below).

For the evaluation of the cross section $\sigma_{\mathrm{PI}}^{\mathrm{el}}$ (cf. Supplementary Fig. 6b), we employ the projector augmented wave method as implemented in the VASP package [7, 8] and the PBE exchange-correlation functional [9]. The defect is modeled using a hexagonal 6×6×2 supercell with 576 lattice sites. The Brillouin zone is sampled by a 15×15×15 k-point grid centered at the Γ point following [5]. For the geometry optimisation of the ground state and the first excited quartet state (within constrained density functional theory), the calculation of ionisation levels, and to generate the starting point for calculations with the CI-CRPA method (cf. [3, 6] for details) we employed the same supercells, the hybrid functional HSE [10, 11], and a single k-point (Γ). For the description of the wave function a cut-off energy for of 420 eV for the plane-waves and of 840 eV for the augmentation is used. ionisation energies are calculated using charge state cor-

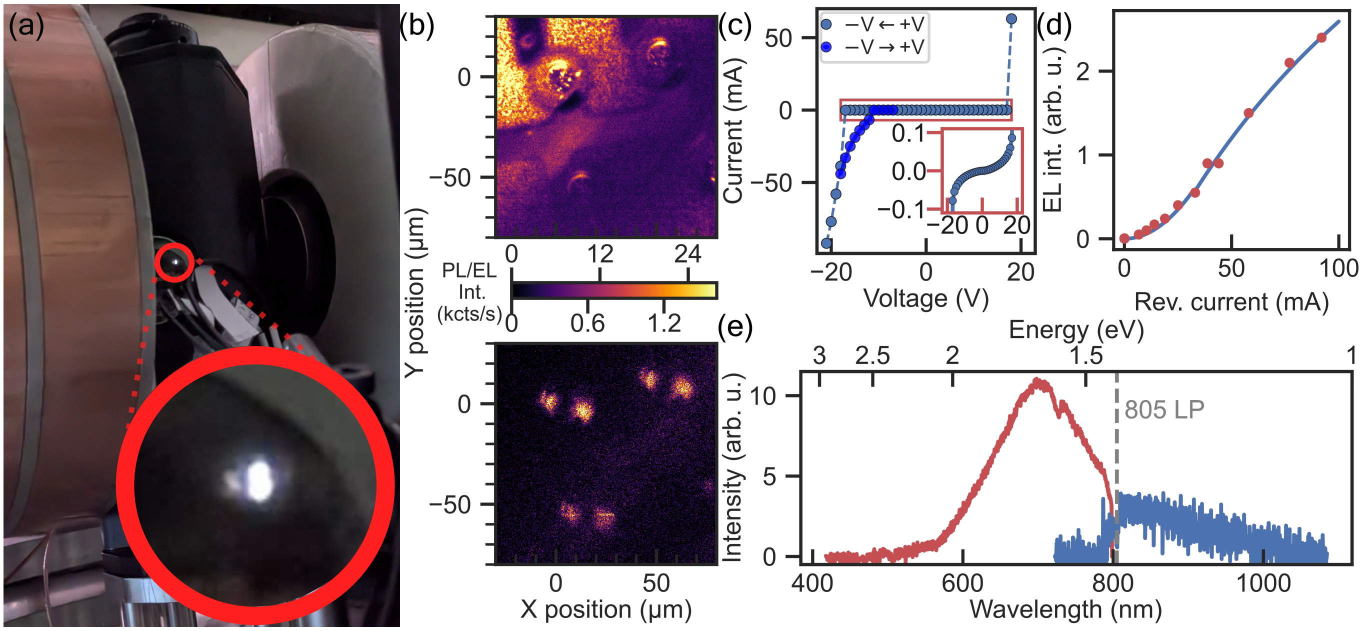

SUPPLEMENTARY FIGURE 5. Electroluminescence in 4H-SiC at ∼10 K: **(a)** Picture of the Montana Cryostat, where the light emitting sample can be seen by pure eye. The luminescence appear white. **(b)** Confocal maps measured with PL and EL. The light emitted sidewise from the sample is reflected internally and is propagated to the objective after reflection at the SIL surfaces. **(c)** UI characteristics of contact 1 shows diode behaviour in both directions, positive and negative. For voltages beyond the threshold sudden jump in current can be observed. Sample heating up to 14 K is caused by current flow. Dark blue curve shows hysteresis while going back to 0 V. Red colored inset shows the zoomed curve inside of the red rectangular. **(d)** EL intensity measured with the reflection signal on the upper left SIL. Intensity signal is approximated using first an exponential growth followed by a saturation. **(e)** Spectra of the EL light, measured with two spectrometers on each side of the fixed 805 nm LP filter. Shows really broad spectrum around 700 nm.

rections (see [3]). We obtain the spectral function $A(\varepsilon)$ (cf. the inset in Fig6b) required for the full cross section $\sigma_{\mathrm{PI}}(\varepsilon)$ via the generating function approach (e.g. [12]). The reference configurations for the initial and final states are the equilibrium geometries of the first excited quartet V_{Si}^{-} and the ground state of V_{Si}^{2-} as in the final state the interaction of the free hole with the defect should be negligible. We perform the relaxation with the HSE exchange-correlation functional, in the former case within the constrained density functional theory and the occupation scheme depicted in Supplementary Fig. 6a. For the calculation of the vibrational spectrum, we employ the supercell embedding technique for the ground state of V_{Si}^{-} and based on a 13×13×13 supercell and cutoffs $r_1 = 7.775$ Å and $r_2 = 7.77$ Å for the defect and bulk force constants, respectively. Force constants are calculated for a smaller 5×5×2 supercell, a 2×2×2 Γ-centered k-point set, and the PBE [9] exchange-correlation functional. The bulk contributions are calculated with the PHONOPY package [13, 14]. To reduce the high computational costs associated with the defect cells, we employ the HIPHIVE toolkit [15, 16] to fit the force constants to 65 rattled reference structures with the least-squares method. Our model achieves an RMSE of 9.2 meVÅ^{-1} when evaluated on a holdout test set containing 5 % of the dataset.

The theoretical power-dependent photon absorption rate Γ_{PI} shown in (Fig. 3d) of the main text is obtained by multiplication of the cross section σ_{PI} with the impinging photon flux Φ divided by the laser power P, i.e. $\Gamma_{\mathrm{PI}}(\lambda) = \sigma_{\mathrm{PI}}(\frac{hc}{\lambda})\,\frac{\lambda}{hc\,A_{\mathrm{spot}}}$, where $A_{\mathrm{spot}} = \pi\,(0.9\lambda/N_{\mathrm{A}})^2$ is the size of the laser spot for a numerical aperture of the focusing lens $N_{\mathrm{A}} = 0.75$. Note that Γ_{PI} in contrast to the measured two-photon ionisation rate does not include the quantum efficiency of the resonant PLE as well as competing relaxation processes that deplete the occupation of the first excited state. Such processes include spontaneous emission and spin-selective non-radiative relaxation via inter-system crossing and metastable states. In Fig. 3d, we therefore show Γ_{PI} divided by its maximum value of $\Gamma_{\mathrm{PI}}^{\max}$ =27 873.13 Hz µW^{-1}.

V. SUPPLEMENTARY NOTE 5: THE PHOTOIONISATION OF THE V_C IN 4H-SIC

The calculation is performed using VSAP within the density functional theory (DFT) framework with the Heyd-Scuzeria-Ernzerhof (HSE) hybrid functional and related HSE06 parameters [18–20]. The carbon vacancy at the cubic lattice site, i.e., the V_C(k) is also modeled in a 576-atom 4H-SiC supercell (6x6x2) with a Brillouin-zone of Γ-point sampling. We optimised the atomic geometry of

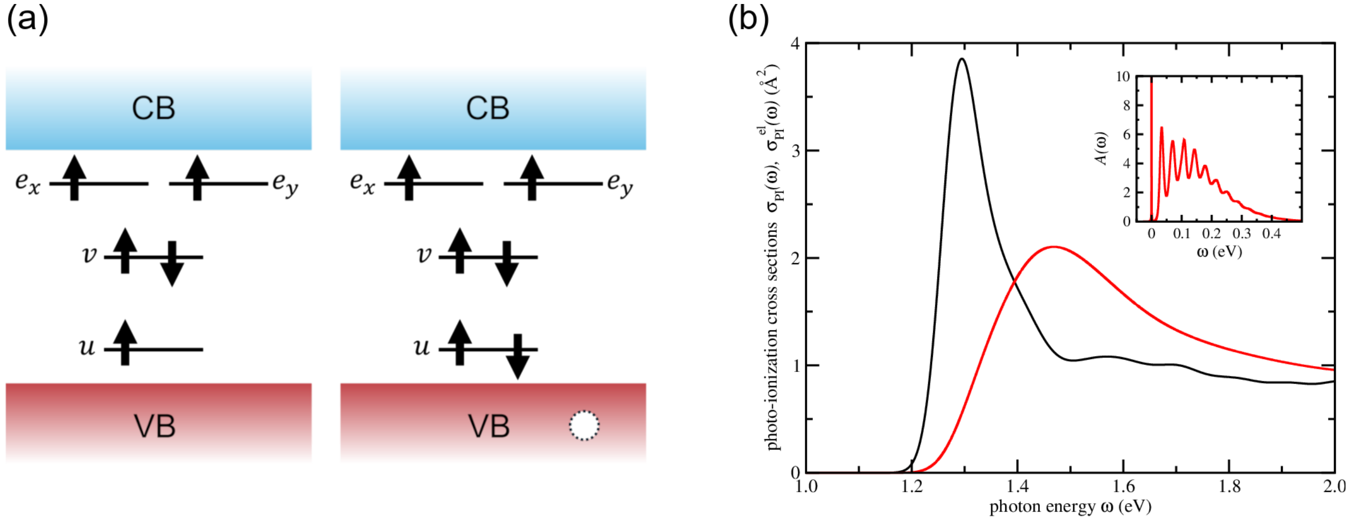

SUPPLEMENTARY FIGURE 6. **(a)** Level schemes of the initial first excited quartet (V_{Si}^{-}) and final ionised states ($V_{Si}^{2-}+h^{+}$). In the PLE the excited quartet is produced from the groundstate by promoting an electron from the u to v level. The second photon excites a valence band electron to the now empty u level, generating the ground state of V_{Si}^{2-} plus a hole. **(b)** Photo-ionisation cross section σ_{PI}^{el} (black) and σ_{PI} (red). The spectral function between the first excited state and the ground state of V_{Si}^{2-} used in the convolution (see Eq. 2) is shown in the inset.

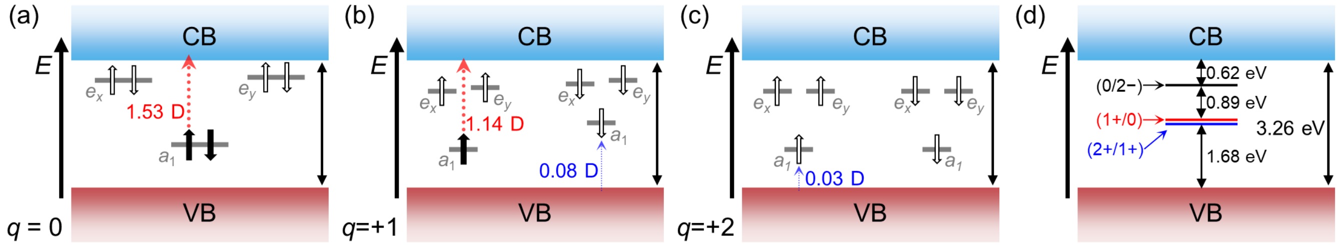

SUPPLEMENTARY FIGURE 7. **(a)-(c)** Sketch of the single particle energy level structure of V_C(k) under charge states of $q = 0$, $q = +1$, and $q = +2$, respectively. The black solid single arrow represents energy hierarchy; the gray horizontal line denotes the local single-particle energy level occupied by electrons (solid arrows) or holes (hollow arrows) with spin; the dashed lines indicate dipole transitions, and the double arrows represent energy gaps. **(d)** Charge state transition level picture ofV_C(k) with data taken from our previous work [17].

the V_C(k) in three charge states: $q = 0$ (neutral), $q = +1$, and $q = +2$. We took into account the possible existence of the Jahn-Teller (JT) effect during the geometry optimisation process. We find both the neutral and $q = +1$ charge states possess the JT distortion, which causes the lifting of degeneracy in the energy levels, as shown in Figure 7a and b. V_C(k) with a charge state of $q = +2$ keeps the high symmetry of C_{3v} unchanged. We calculate the neutral V_C(k) center by non-spin-polarised DFT because of the close shell of the electron structure, while the other two charge states are spin-polarised. The transition dipole moment (TDM) between localised states and conduction (valance) bands is calculated using the VASP-BandUnfolding package [21] to reveal the probability of ionisation of V_C(k) center, which may affect the charge environment around the vicinity of the V2 center. From Figure 7a and b, For both neutral and $q = +1$ charge states, excitation from localised states to the CB dominates, with a much larger TDM than excitation from the VB to localised states (only in $q = +1$). The charge state transition energies are as shown in Supplementary Fig. 7d. For $q = +2$, there only exists an excitation from VB.

VI. REFERENCES

[1] D. Liu, F. Kaiser, V. Bushmakin, E. Hesselmeier, T. Steidl, T. Ohshima, N. T. Son, J. Ul-Hassan, Ö. O. Soykal, and J. Wrachtrup, The silicon vacancy centers

in sic: determination of intrinsic spin dynamics for integrated quantum photonics, npj Quantum Information **10**, 72 (2024).

[2] Comsol multiphysics® v. 6.2. www.comsol.com. comsol ab, stockholm, sweden.

[3] M. Widmann, M. Niethammer, D. Y. Fedyanin, I. A. Khramtsov, T. Rendler, I. D. Booker, J. Ul Hassan, N. Morioka, Y.-C. Chen, I. G. Ivanov, et al., Electrical charge state manipulation of single silicon vacancies in a silicon carbide quantum optoelectronic device, Nano letters **19**, 7173 (2019).

[4] G. de Lange, Z. H. Wang, D. Ristè, V. V. Dobrovitski, and R. Hanson, Universal dynamical decoupling of a single solid-state spin from a spin bath, Science **330**, 60 (2010).

[5] L. Razinkovas, M. Maciaszek, F. Reinhard, M. W. Doherty, and A. Alkauskas, Photoionization of negatively charged nv centers in diamond: Theory and ab initio calculations, Physical Review B **104**, 235301 (2021).

[6] M. Bockstedte, F. Schütz, T. Garratt, V. Ivády, and A. Gali, Ab initio description of highly correlated states in defects for realizing quantum bits, npj Quantum Materials **3**, 31 (2018).

[7] G. Kresse and J. Furthmüller, Efficient iterative schemes for ab initio total-energy calculations using a plane-wave basis set, Physical review B **54**, 11169 (1996).

[8] G. Kresse and D. Joubert, From ultrasoft pseudopotentials to the projector augmented-wave method, Physical review b **59**, 1758 (1999).

[9] J. P. Perdew, K. Burke, and M. Ernzerhof, Generalized gradient approximation made simple, Physical review letters **77**, 3865 (1996).

[10] J. Heyd, G. E. Scuseria, and M. Ernzerhof, Hybrid functionals based on a screened coulomb potential, The Journal of chemical physics **118**, 8207 (2003).

[11] A. V. Krukau, O. A. Vydrov, A. F. Izmaylov, and G. E. Scuseria, Influence of the exchange screening parameter on the performance of screened hybrid functionals, The Journal of chemical physics **125**, 224106 (2006).

[12] L. Razinkovas, M. W. Doherty, N. B. Manson, C. G. Van de Walle, and A. Alkauskas, Vibrational and vibronic structure of isolated point defects: The nitrogen-vacancy center in diamond, Physical Review B **104**, 045303 (2021).

[13] A. Togo, L. Chaput, T. Tadano, and I. Tanaka, Implementation strategies in phonopy and phono3py, J. Phys. Condens. Matter **35**, 353001 (2023).

[14] A. Togo, First-principles phonon calculations with phonopy and phono3py, J. Phys. Soc. Jpn. **92**, 012001 (2023).

[15] F. Eriksson, E. Fransson, and P. Erhart, The hiphive package for the extraction of high-order force constants by machine learning, Advanced Theory and Simulations **2**, 1800184 (2019).

[16] E. Fransson, F. Eriksson, and P. Erhart, Efficient construction of linear models in materials modeling and applications to force constant expansions, npj Computational Materials **6**, 135 (2020).

[17] Krisztián Szász, Viktor Ivády, Igor A. Abrikosov, Erik Janzén, Michel Bockstedte, and Adam Gali, Spin and photophysics of carbon-antisite vacancy defect in 4h silicon carbide: A potential quantum bit, Physical Review B **91**, 121201 (2015).

[18] J. Heyd, G. E. Scuseria, and M. Ernzerhof, Hybrid functionals based on a screened coulomb potential, The Journal of Chemical Physics **118**, 8207 (2003).

[19] A. V. Krukau, O. A. Vydrov, A. F. Izmaylov, and G. E. Scuseria, Influence of the exchange screening parameter on the performance of screened hybrid functionals, The Journal of Chemical Physics **125**, 224106 (2006).

[20] P. Deák, B. Aradi, T. Frauenheim, E. Janzén, and A. Gali, Accurate defect levels obtained from the hse06 range-separated hybrid functional, Physical Review B - Condensed Matter and Materials Physics **81**, 153203 (2010).

[21] Q. Zheng, https://github.com/qijingzheng/ vaspbandunfolding (2019).
